# Cardiovascular and lipid-lowering effects of a marine lipoprotein extract in a high-fat diet-induced obesity mouse model

**DOI:** 10.7150/ijms.80727

**Published:** 2023-01-22

**Authors:** Iván Carrera, Lola Corzo, Vinogran Naidoo, Olaia Martínez-Iglesias, Ramón Cacabelos

**Affiliations:** EuroEspes Biomedical Research Center, Institute of Medical Science and Genomic Medicine, 15165-Bergondo, Corunna, Spain.

**Keywords:** Cardioprotective, Obesity, Cardiovascular disease, Marine lipoproteins, Nutraceutical

## Abstract

Obesity is a major health challenge worldwide, with implications for diabetes, hypertension and cardiovascular disease (CVD). Regular consumption of dark-meat fish is linked to a lower incidence of CVD and associated metabolic disorders due to the presence of long-chain omega-3 fatty acid ethyl esters in fish oils. The aim of the present study was to determine whether a marine compound like a sardine lipoprotein extract (RCI-1502), regulates fat accumulation in the heart of a high-fat diet-induced (HFD) mouse model of obesity. To investigate its effects in the heart and liver, we conducted a randomized, 12-week placebo-controlled study in which we analyzed the expression of vascular inflammation markers, obesity biochemical patterns and related CVD pathologies. Male HFD-fed mice treated with a RCI-1502-supplemented diet showed reduced body weight, abdominal fat tissue and pericardial fat pad mass density without systemic toxicity. RCI-1502 significantly reduced triacylglyceride, low-density lipoprotein and total-cholesterol concentrations in serum, but increased HDL-cholesterol levels. Our data show that RCI-1502 is beneficial for reducing obesity associated with a long-term HFD, possibly by exerting a protective effect on lipidic homeostasis, indicated also by histopathological analysis. These results collectively indicate that RCI-1502 acts as a cardiovascular therapeutic nutraceutical agent, which modulates fat-induced inflammation and improves metabolic health.

## Introduction

Obesity is a risk factor for various cardiovascular disease (CVD) subtypes, including coronary heart disease (CHD), heart failure, and stroke. These pathologies are linked to hypertension 1,2 and low-grade inflammation 3, considered to be the primary mechanisms behind the adverse effects of obesity 4-6. High levels of low-density lipoprotein triacylglycerol (LDL-TG) dysregulate high-density lipoprotein (HDL) metabolism, by increasing cholesteryl ester transfer protein levels and promoting the breakdown of TG-rich HDL in serum 7. The accumulation of TG-rich lipoproteins, therefore, plays a major role in atherogenesis, including foam cell formation, endothelial and vascular inflammation 8. Hypertriacylglycerolemia, a condition related to abnormal lipid metabolism, is associated with visceral obesity and is a risk factor for coronary artery disease 9-11. Although the metabolic mechanism is not fully understood, omega-3 fatty acid ethyl esters (ω-3 FAEEs) reduce LDL-TG levels in serum, preventing dysregulation associated with hypertriacylglycerolemia 12,13. The two main omega-3 FAEEs from fish oil, krill oil or marine microalgae are the eicosapentaenoic acid (EPA) and the docosahexaenoic acid (DHA). Both of them are linked to beneficial cardiovascular clinical effects, such as the increasing hepatic flux of fatty acids from dietary sources 14, lowering blood triglycerides 15 and total cholesterol 16, inhibiting LDL-C oxidation 17, protecting endothelial function and reducing hepatic secretion of LDL-apoB-100 in serum 18 which may be effective for treating obesity-related dyslipidemia.

Fatty liver disease (FLD), which is closely associated with obesity, is linked to a variety of extrahepatic metabolic syndrome (MetS)-linked diseases, such as type 2 diabetes and CVD 1,2,19. In individuals with obesity, there is a strong correlation between increased hepatic fat content and subclinical atherosclerosis and cardiac function alterations, besides other risk factors 20,21. The prevalence of MetS and pre-diabetes pathology is increased in patients with FLD; this may be a determinant factor of liver impairment (liver fibrosis, hepatic inflammation, and liver cell death) and adipose insulin sensitivity 22. A decrease in the mitochondrial fatty acid beta-oxidation rate, deficient intake of triglycerides as LDL, an exponential increase in endogenous fatty acid synthesis or enhanced delivery of fatty acids to the liver 23 are all potential pathophysiologic mechanisms in FLD.

Obesity-induced inflammatory mechanisms are complex, involving several cellular components and mediators 1. A prolonged high-fat diet (HFD) in mice increases adiposity, causes dysregulation of systemic T helper type 1 cells by altering the Th1/Th2 cell ratio, and induces secretion of inflammatory cytokines, such as monocyte chemoattractant protein 1 (MCP-1), tumor necrosis factor alpha (TNF-α) and interleukin 1-beta (IL-1β) 4,6. These processes, and the huge infiltration of natural killer- and cytotoxic CD8+ T cells into adipose tissue are primarily responsible for macrophage recruitment and a state of chronic inflammation in obese mice 24.

The current study aims include not only contributions to new knowledge of pathological effects of obesity on mice metabolism, but also the use of a new natural marine bioactive compound capable of modulating cardiovascular risk and the related immune response 25-30. Here we hypothesized that supplementing mice on a high-fat diet with RCI-1502 (HFD/CS mice) would decrease their TC and LDL levels since it contains 60-80% lipoproteins and polyunsaturated fatty acid (EPA and DHA content) 25,31. The main strength of this study is the wide range of experimental analysis focused on the metabolic syndrome features by using multi-technical approaches such as immunological markers, biochemical determinations and behavioral evaluation. This preclinical HFD animal models seem to mimic the mechanisms that induce obesity and MetS in humans 32; and thus, it's used in this study for preclinical testing in order to address the role of diet, etiology, pathophysiology and possible future therapeutic interventions.

## Materials and Methods

### Ethics statement

All experimental procedures were performed in accordance with the European Community Law (86/609/EEC), European Union Directive 2016/63/EU and the Spanish Royal Decree (R.D. 1201/2005) and were approved by the Ethics Committee of the EuroEspes Research Center (permit number: EB/2015-037).

### Experimental animals

Male wild-type C57BL/6 mice (n = 54) were bred from colonies, established from mice originally donated by Dr. M. Lakshmana (Florida International University, USA). The mice were housed under a 12 h light/12 h dark cycle in a room at 22 ± 0.5 °C with 40-50% humidity, and had free access to food and water. Based on data obtained in previous studies concerning the sex-dimorphic effect on obesity response and in order to assure a minimum variation on the baseline-obesity timeline in HFD mice, we use only males in this study.

### Preparation of the marine lipoprotein extract (RCI-1502)

RCI-1502, a lipoprotein complex of marine origin, was extracted from the muscle of the marine species *S. pilchardus* (European sardine, Clupeidae family) by non-denaturing biotechnological processes, which preserves the natural properties of its active ingredients. This patent process is based on the lyophilization method, in which water is removed from a product after it is frozen and placed under a vacuum, allowing the ice to change directly from solid to vapor without passing through a liquid phase. RCI-1502 has a high protein content and saturated fatty acids (mainly palmitic acid), monounsaturated fatty acids (primarily oleic and palmitoleic acids), polyunsaturated fatty acids (mainly eicosapentaenoic, eicosatetraenoic, and docosahexaenoic acids); it is also rich in vitamins (principally B5 and C) and minerals (mainly potassium, phosphorus and calcium), as detailed in Table 1.

RCI-1502 was prepared as pellet biscuits by combining RCI-1502 powdered extract (50%) with diet wheat and Milli-Q water (10%; Millipore), and left to dry overnight at 34 °C. The food pellets were stored in air-tight containers at 4 °C.

### High-fat diet (HFD)-induced mouse model of obesity

The addition of corn oil (composed of 99% triacylglycerol, with polyunsaturated fatty acid (PUFA) 59%, monounsaturated fatty acid 24%, and saturated fatty acid (SFA) 13%) to the food pellets represented the HFD supplementation vehicle, although its ad libitum ingestion does not allow exact control of the dose that each animal receives, only a percentage in relation to the total ingested in the diet. To avoid possible rancidity and oxidation of the compounds researchers performed daily feed exchanges. The eight-week supplementation based on oil administration included in diet (Table 2 and 3), induced a ranged from 50 to 60% of kcal from fat, while in the control groups, they ranged from 10 to 15%. According to recent C57BL/6 mice studies where obesity was induced, an hypercaloric diet with almost 60% of the calories being from lipids are considered effective in promoting obesity and metabolic changes associated with the disease 33. The diet/supplementation regimen (Table 2), according to the nutritional composition of the different diets (Table 3) was performed during eight weeks. Male mice (n = 54; 6-8 weeks old) were randomly divided into six groups of nine animals each, according to the following supplementations: (1) Group (Gr) A: High-fat diet before and after supplementation with RCI-1502 (HFDba); (2) Gr B: High-fat diet after supplementation with RCI-1502 (HFDa); (3) Gr C: High-fat diet before supplementation with RCI-1502 (HFDb/RCI-1502); (4) Gr D: Normal diet before and after RCI-1502 mice supplementation, as toxicity control (Diet); (5) Gr E: High-fat diet only (HFD); and (6) Gr F: Normal diet as negative control. Male mice with body weights <15 g or >25 g at six-weeks of age, were not included in the study.

The same, measured, quantities of food pellets were administered to control and treated mice. In order to show that the amount of HFD reached the minimum obesity biochemical parameters and so to be compared among the different HFD-groups, a quantification study was performed as a pilot experiment (Supplementary data). The health of the animals was monitored twice a day, and their weights assessed once per week.

### Blood collection and sample preparation

At the end of the experimental period, serum was collected directly from the ventricle, centrifuged at 4.500 rpm, the blood serum was collected and stored at -80 °C. This cardiac puncture method is a suitable technique to obtain a single, large, good quality sample from a mouse under deep terminal anesthesia (intraperitoneal injection of Avertin (Sigma-Aldrich), as required for metabolic, lipidic, hepatic, nutritional, oxidative and cardiovascular biomarker measurements. Animals under anesthesia were transcardially perfused with 0.9% NaCl followed by 4% paraformaldehyde (PFA, Cat. #43368 Alfa Aesar™, Germany). Hearts and livers were removed and placed into 4% PFA for 48 h. The tissues were then immersed in 0.1 M phosphate buffer (PB, pH 7.4) for 12 h, cryoprotected with 30% sucrose in PB, embedded in optimal cutting temperature (OCT) compound (Tissue Tek, Torrance, CA) and frozen with liquid nitrogen-cooled isopentane. Parallel series of transverse sections (18 μm-thick) were cut on a cryostat, mounted on Superfrost Plus slides (Menzel Glasser, Madison, WI), and stored at room temperature for histopathological analysis.

### Histology and immunohistochemistry

Routine histology was performed on Sudan Black (SB) and hematoxylin and eosin (HE)-stained sections to identify and characterize morphological changes that occur during the cardiovascular disease process, such as vacuolar degeneration, major myofibril changes, adiposomes, and lipid vacuolization 28. To detect the expression of complementary cardiovascular histopathological markers we used immunohistochemistry. Commercially available antibodies were used, such as mouse monoclonal anti-VCAM-1 (1:400; Affymetrix eBioscience) in heart samples.

To eliminate endogenous peroxidases, sections of heart and liver were pretreated with H_2_O_2_ in phosphate-buffered saline (PBS, pH 7.4) at 37 °C for 15 min. The tissues were rinsed twice in 0.05 M tris-buffered saline (TBS) containing 0.1% Tween-20 (TBS-T, pH 7.4) for 10 min each, and endogenous avidin-binding activity blocked (Vector kit) for 30 min. The sections were incubated overnight at 4 °C with MOM blocking buffer and primary antibodies previously described. The mouse-on-mouse peroxidase immunodetection system (MOM Kit; Vector) was used to eliminate any nonspecific binding of anti-mouse secondary antibodies with the endogenous mouse immunoglobulins in the tissue, according to the manufacturer's instructions. The sections were rinsed in TBS-T (three times for 10 min), incubated in goat anti-rabbit (Dako) or goat anti-mouse (Dako) secondary antibodies for 1 h at room temperature, washed in TBS-T (three times for 10 min) and then incubated for 30 min with the avidin-biotin-peroxidase complex (Vectastain; Vector). Signal detection was performed with 3,3-diaminobenzidine (Sigma-Aldrich) as the chromogen and hydrogen peroxide as the oxidant. Negative controls were included by omitting the primary or secondary antibodies. Sections were then dehydrated in a graded series of ethanol washes, and coverslipped with a rapid embedding agent (Eukitt; Fluka). Images were visualized on a microscope (Olympus BX50) and digital images obtained (DP-10; objective 10x lens, Olympus). The photographs were adjusted only for brightness and contrast with Corel Photo-Paint (Corel, Ottawa, Canada). Immunohistochemical hallmarks were classified as positive for VCAM-1 if cytoplasmic staining was detected by using area/pixel analysis software (Pixcavator 4) to quantify the number of pixels inside the outer boundary of each cell body; this aided quantification of the density of immunofluorescence cell markers relative to background. Two different observers independently evaluated the experimental group slides in a double-blinded manner; both achieved a high level of concordance.

### Locomotor activity and body mass percentage

**Figure 1:**
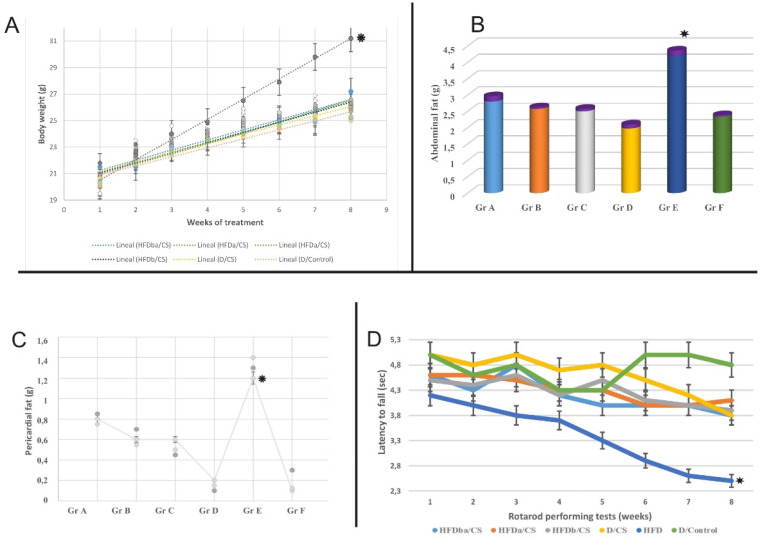
** Progressive quantification of body weight gain, abdominal fat, pericardial fat and rotarod performance of treated mice.** The diets used were normal control diet (control; Gr F), high-fat diet (HFD; Gr E), Normal diet before and after RCI-1502 (D/CS; Gr D), HFD before RCI-1502 (HFDb/CS; Gr C), after (HFDa/CS; Gr B) or both (HFDba/CS; Gr A) in mice for 8 weeks. **A-C,** The bars represent the means and SEM of mice per diet group in measured weight gain and fat (indicated in grams). Significant differences of HFD group from control and HFD/CS groups were addressed by statistical analysis (p<0.05, Mann Whitney U-Test with Bonferroni's correction). Standard deviation is indicated in purple or in bars. **D,** Motor coordination efficiency of HFD/CS groups was significantly improved on rotarod performance when compared with HFD treated group (*p < 0.05).

To examine the effects of HFD and RCI-1502 consumption on mice behavior, a rotarod apparatus (IITC Life Science, Inc., Woodland Hills, CA) was used to assess motor coordination and balance 34. The main reason for evaluating the locomotor activity was to address the correlation between the beneficial of the CS supplementation and the motor coordination/agility, in order to reinforce the data of the health effects on this obesity mice models. The instrument consists of a circular rod turning at a constant speed (15 rpm) where animals will try to remain on the rod rather than fall, while the time of fall is recorded. The rotarod accelerated from 1 rpm to 15 rpm over 90 sec, with each trial again lasting a maximum of 120 sec. Trials ended when mice either fell off the rod or clung to the rod as it made two complete rotations. To achieve a basal level of performance, each mouse underwent three days of training prior to injury, in which three trials per day conducted, with a 10 min interval between trials. The rotarod test was performed at the end of each week. Individual scores from three trials were averaged and evaluated relative to their baseline latencies (Fig. 1).

The body mass index of each experimental mouse was determined by calculating the mass (g)/height (cm) ratio. The abdominal and pericardial fat percentages were determined by removing and weighing the fat depots around the abdominal cavity and the heart, following euthanasia.

### Serum lipids and specific cardiovascular markers

After sacrifice, blood serum was collected from each group of mice for biochemical analysis (Table 4). The quantification of serum biomarkers related to renal function (urea and creatinine), vascular risk [triglycerides (TG), High-density lipoprotein (HDL) cholesterol, Low-density lipoprotein (LDL) cholesterol, total cholesterol (TC), homocysteine (HCY)], hepatic injury [glutamate oxaloacetate transaminase (GOT) and glutamine-pyruvate transaminase (GPT)], nutritional status (albumin) and antioxidant status (TAS) was obtained by using commercial reagents at the automated UV-visible spectrophotometer Cobas Mira Plus Analyzer (ROCHE Diagnostics, Basel, Switzerland). Complementary vascular markers [lipoprotein (a) (Lpa) and hypersensitive-C reactive protein (hs-CRP)] were analyzed from blood serum using the commercial mouse-specific ELISA kits (MyBiosurce, San Diego, USA and Elabscience, Hubei, China, respectively). Quantification was conducted using the best-fit equation of standards in Curve Expert software 2.5.

### Histamine determinations

Liver samples for histamine determination were homogenized in 0.5 mL perchloric acid (2%) by ultrasonic cell disruption. Sample homogenates were centrifuged at 12,500 rpm for 30 min. The pellet was discarded and the supernatant was stored at -40 °C for analysis. Histamine levels were measured by high performance liquid chromatography (HPLC) using a stainless-steel column packed with a cation exchanger (TSKgel SP-2SW, 5 μm particle size) and automated Shore's OPA fluorometric detection system equipped with a chromatographic system (Agilent 1100 series). The fluorescence intensity was measured at 450 nm with excitation at 360 nm in a spectrofluorometer (Agilent 1100 series). Under these conditions the retention time for histamine was 14.0±1.0 min. Homogenized liver samples (20 μL) were injected directly into the HPLC column. The minimum detection limit of the system was 0.05 pmol, and the intra- and inter-assay coefficients of variations were 2-6% and 7-11%, respectively. Substances causing interference in Shore's OPA reaction, such as ammonia, histidine, spermine, and spermidine, were separated in the column; as their fluorescence intensities were short, histamine was determined by injecting a perchloric acid tissue extracts directly into the column eliminating the need for prior purification.

### Statistical analysis

Statistical analysis was performed using SPSS software (Version 23.0, Chicago, USA). Data were tested for normality using Shapiro-Wilk test. Differences between treated groups were compared with the Kruskal-Wallis test followed by the Mann-Whitney U-test. Data are presented as standard error of the mean (SEM); a *p value <0.05 indicates statistical significance.

## Results

### RCI-1502 reduces the body weight of HFD mice without compromising locomotor activity

The HFD caused a significant increase in cumulative body weight of mice (*p* < 0.05) when compared with controls and other treated groups. Mice body weight significantly increased (*p* < 0.05) due to the high-fat diet, with a cumulative body weight gain (Fig. 1A). RCI-1502-supplemented HFD mice (Gr A-C) had significantly reduced body weight (Fig. 1A-C). The mean cumulative body weight gain over eight weeks was 20-25% lower in HFD/CS mice (*p* < 0.05) and 25-30% lower in diet/CS mice (*p* < 0.01) than in HFD mice (Fig. 1A-C). Quantification data from the abdominal (Fig. 1B) and pericardial fat pads (Fig. 1C) exhibit a clear correlation, where the HFD group notably show a significant difference compared with the other experimental groups. We tested the hypothesis that the anthropometrical body mass index (BMI) may identify obesity and may predict its adverse effects on lipid profile in mice. The results showed that after 8 weeks of dietary supplementation, the final body weight, body mass gain and BMI were higher in HFD mice than in any other experimental group. The BMI reached the highest value in HFD at 8 weeks of supplementation (3.39 ± 0.3), correlated with a large increase in body weight (∼25%), in comparison with control group (2.57 ± 0.02) (*P* < 0.01), indicating a clear pattern of obesity in HFD treated group.

Locomotor activity remained the same between the HFD-CS groups; however, mice on the HFD diet displayed significantly less locomotor activity during the entire study period than RCI-1502 (Gr A-C) and control animals (Fig. 1D; *p* < 0.05). Furthermore, HFD-treated mice showed poor locomotor activity and motor coordination than RCI-1502 and control treated mice. However, rotarod performance was not significantly different between HFD/CS and control mice (Fig. 1D).

### RCI-1502 improve the serum lipid profiles of HFD mice

We quantified serum biomarkers related to renal function, vascular risk, hepatic injury, nutritional status and antioxidant capacity in all groups of mice (Table 4). HFD-treated mice had significantly higher levels of most of these biomarkers versus RCI-1502 (CS) and control mice. Among the HFD-CS groups, there was a notable difference in the serum levels of renal function, vascular risk, hepatic injury and antioxidant parameters (as indicated in blold at table 4). Compared to HFD-treated mice, RCI-1502 treated group A (HFDba/CS) showed a significantly low levels of vascular risk parmeters such as HCY (4.1 µmol/L; p<0.05), Lp(a) (1.5 ng/mL; *p* < 0.05), HS-RCP (1.5 mg/dL; *p* < 0.05), whereas RCI-1502 treated group B (HFDa/CS) showed a significantly low levels of renal function parameters [Urea (36 mg/dL; *p* < 0.05), Creatinine (0.32 mg/dL; *p* < 0.05)], hepatic injury [GOT (97 U/L; *p* < 0.05), GPT (23.7 U/L; *p* < 0.05)] and some vascular biomarkers [LDL-CHOL 8.1 mg/dL; TC/HDL-CHOL 1.6 mg/dL *p* < 0.05)]. The group C (HFDb/CS) showed significant decreased levels of cholesterol biomarkers (TC 97,2 mg/dL; HDL-CHOL 57 mg/dL). Between experimental groups, Cholesterol levels were significantly different between HFD-treated mice fed and RCI-1502-supplemented mice (*p* < 0.05; Table 4).

Serum levels of atherogenic (vascular risk and nutritional biomarkers) and obesity-related parameters (renal function, hepatic injury and antioxidant biomarkers) showed significant differences between HFD mice versus all RCI-1502-treated animals (Table 4; *p < 0.05). Present data show that among the RCI-1502-treated groups (Gr A-C), the beneficial effect of adding RCI-1502 in the diet before and after the HFD period (Gr A) was higher than just before (Gr B) or after (Gr C) the HFD period (Table 4).

### RCI-1502 reduces Cardiac and hepatic lipid accumulation

**Figure 2:**
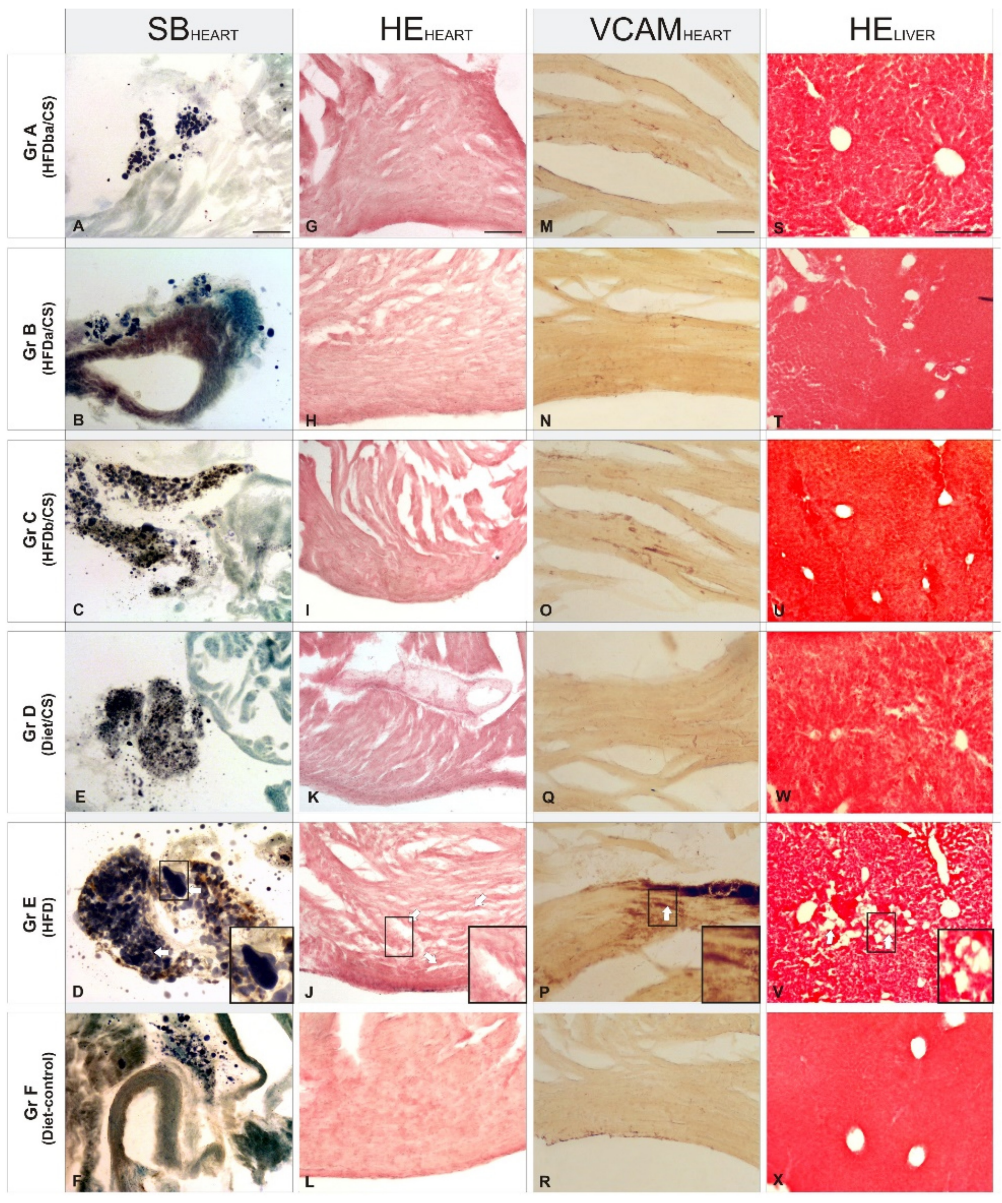
Histopathologic effects in heart and liver sections of RCI-1502 diet supplementation on weight gain and pericardial fat percentage of mice fed with different supplementations.** (A-X)** Representative photomicrographs of pericardial adipose tissue accumulation **(A-F)**, myocardial tissue **(G-L)**, vascular inflammation **(M-R)** and liver vacuolization **(S-X)**. The supplementation diets used were RCI-1502 with high-fat diet before (Group C) and after (Group B) supplementation (Group A), RCI-1502 with normal diet (Group D), high-fat diet (Group E, with magnification inserts) and normal control diet (Group F) for 8 weeks. White arrows show histopathological hallmarks (Groups C and E). Histopathological biomarkers used were VCAM-1 antibodies, hematoxylin-eosin (HE) and Sudan Black (SB), staining in heart and liver tissues. Scale bars: 100μm.

HFD supplementation significantly induced both cardiac and hepatic lipid accumulation revealed by an increase in pericardial fat area, lipid droplet density and liver steatosis (Fig. 2) compared to HFD/CS treated mice (Gr A-C). Histological analysis of pericardial fat pad mass, myocardium and liver tissues in mice supplemented with RCI-1502 (representative of the six supplementation groups) revealed a reduction in cardio- and cerebrovascular pathological hallmarks (Fig. 2).

In Sudan Black B-stained adipose tissue of the pericardium (Fig. 2A-F), there were large and numerous lipid droplets (adiposomes) in HFD-treated mice (Gr E; Fig. 2E), contrasting sharply with the reduced density of small adiposomes in HFD/CS treated mice (Gr A-C; Fig. 2A-C). Among the different HFD/CS supplementations, supplementation of RCI-1502 before and after a HFD feeding regimen reduced the appearance of new adiposomes while maintaining their density. Similar results were observed in mice supplemented with RCI-1502 combined with a normal diet (Gr D; Fig. 2D), although with a reduced density of adiposomes. Hematoxylin and eosin staining in myocardial tissue sections revealed a similar protective pattern of RCI-1502 when supplemented prior to a HFD diet (Gr C; Fig. 2I). Similar lipid accumulation patterns were observed in cardiac tissues of groups A (HFDba/CS) and B (HFDa/CS), where some vacuolar degeneration was observed in myocardial tissue (Fig. 2G,H). However, HFD-treated mice showed severe vacuolar degeneration and massive alteration of myofibrils in the heart (Gr E; Fig. 2K), mainly characterized by the fragmentation of cardiac muscle bundles. This pathologic pattern contrasts with normal myocardial tissue structure in control mice (Gr D and F; Fig. 2J,L), showing homogeneously interconnected, anastomosing, muscle fibers. Moreover, histological examination of the heart in control and RCI-1502-treated mice showed negative or slight immune reactions to VCAM-1 (Gr A-D, and F; Fig. 2M-P,R), a marker of vascular inflammation. HFD-treated mice showed a dramatic increase in VCAM-1 immunoreactivity within the endothelial layers of the heart (Gr E, Fig. 2Q); VCAM-1 staining was notably higher in HFD-treated mice than mice treated with RCI-1502 (Fig. 2). Among the HFD/CS-treated groups (Gr A-C), mice supplemented with RCI-1502 after having been on an HFD-feeding regimen (Gr B), showed less VCAM-I immunoreactive density in the endothelial layer of the heart than in groups A and C (Fig. 2M-R).

**Figure 3:**
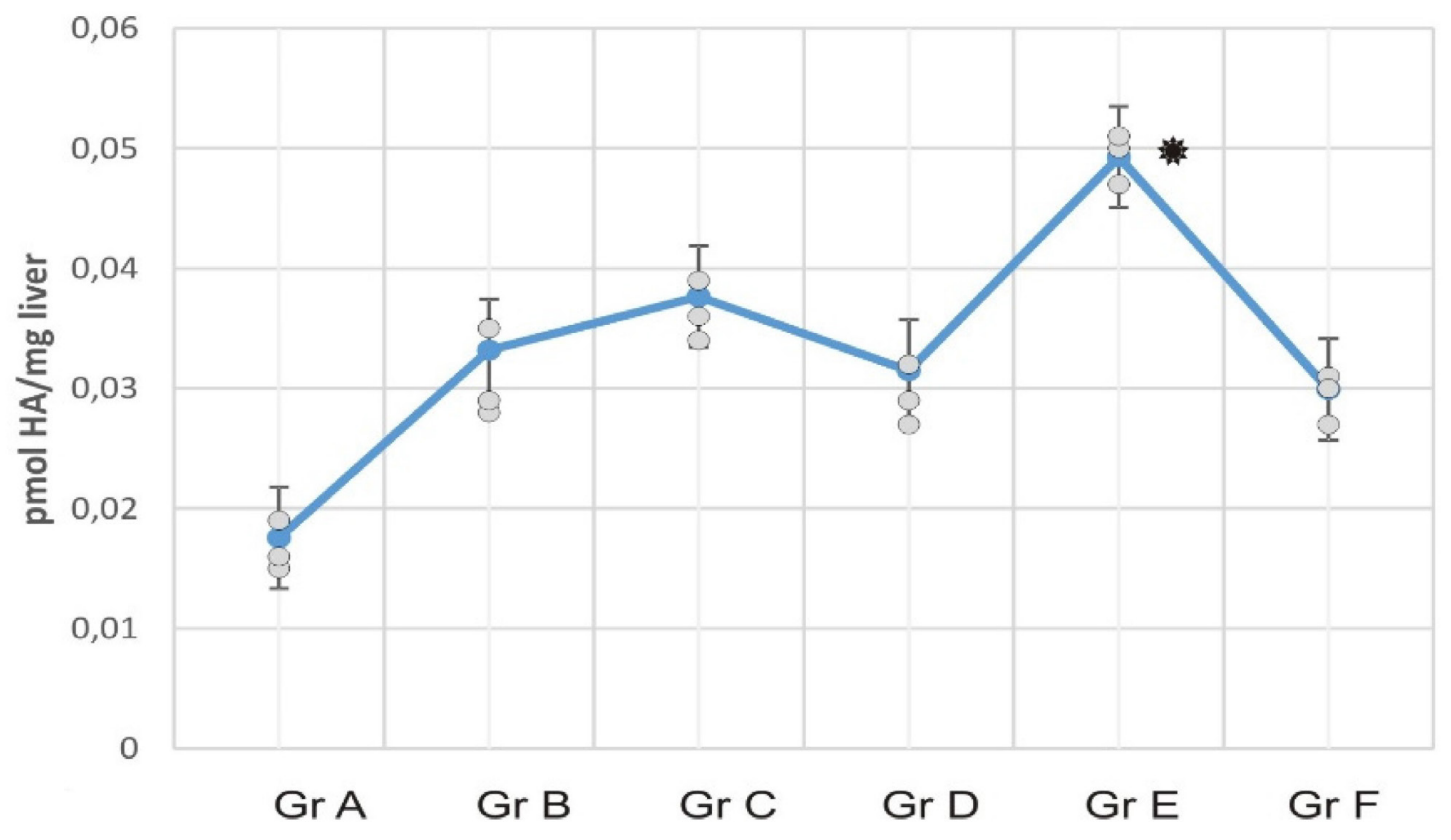
Hepatic levels of histamine in experimental mice groups. Data represent the means ± SEM of liver histamine levels per supplementation group. Note the differences in histamine levels in HFD-fed mice compared with the other HFD/CS groups; histamine levels are significantly reduced in HFDba/CS-treated mice (Grp A).

Histological examination of sections of mice liver (Fig. 2S-X) revealed that controls and HFD/CS-treated mice had normal cellular architecture with radially arranged hepatocytes around the centrilobular vein (Gr. A-C, D and F; Figs. 3S-V,X); however, some lipid vacuolization was observed in HFD/CS groups A and C (Figs. 3S,U). This pattern sharply contrasted with that in sections from HFD-treated mice where a high density of lipid vacuolization, fatty hepatocyte degeneration and disintegration of hepatic cords was found; this indicated liver injury due to the high accumulation of cholesterol (Gr. E, Fig. 2W). Quantification of the mean area of vacuolization among HFD/CS treated groups showed relative higher rates in group B than in groups A and C versus control groups (Figs. 2S-X).

### RCI-1502 reduces histamine accumulation levels in liver

Histamine levels were analyzed in liver mice samples of all mice groups to determine the inflammation data of RCI-1502 in HFD-fed mice (Fig. 3). HFD mice exhibited high histamine concentrations compared with the HFD mice treated with RCI-1502. Statistically significant differences were detected between HFDba/CS and HFD mice groups (Fig. 3; *p < 0.05).

## Discussion

**Figure 4:**
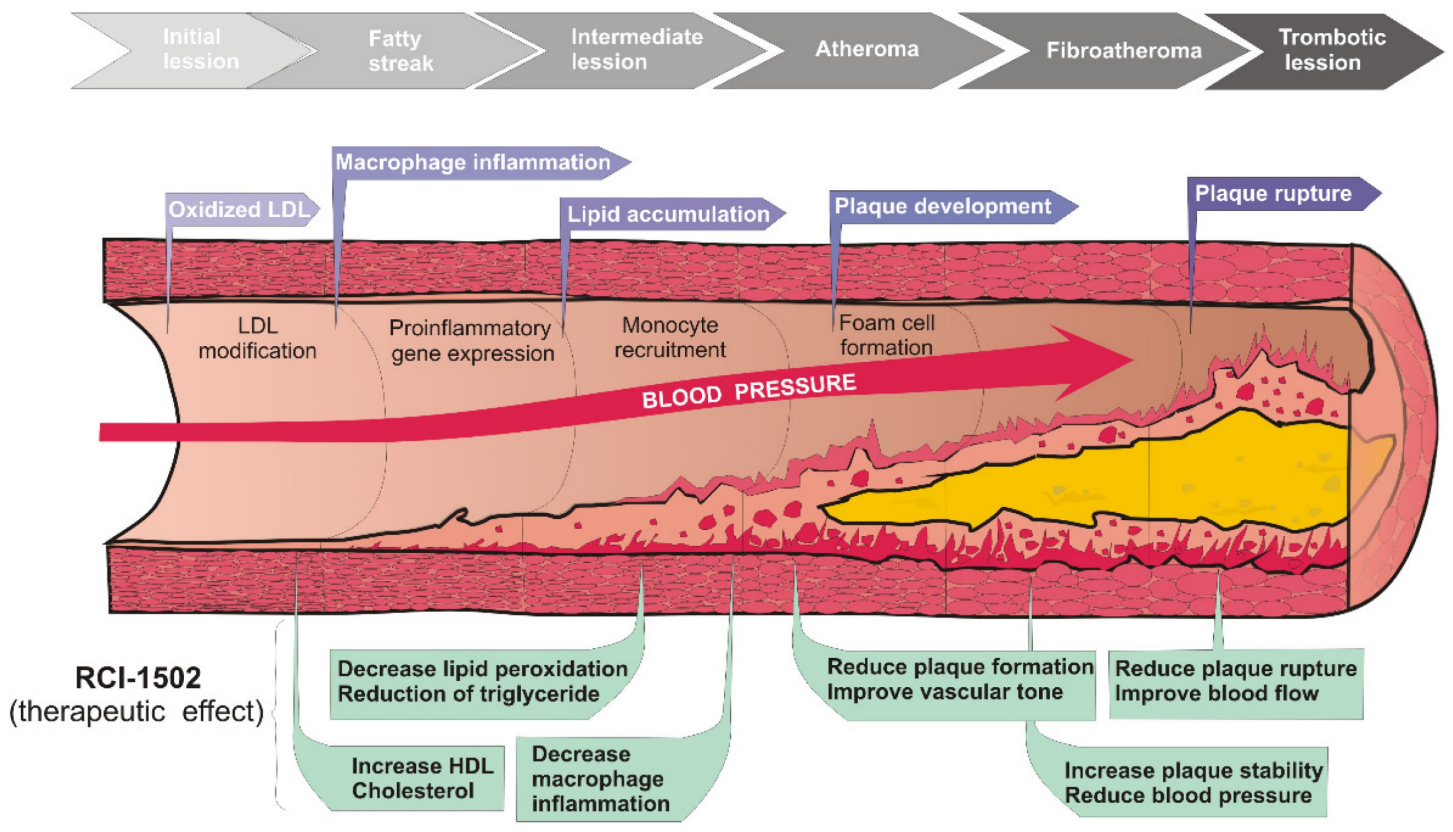
Working model of possible effects of RCI-1502 on prevention of atherosclerosis and related developmental processes.

The aim of this study was whether RCI-1502, a sardine-derived extract, as a part of a HFD, can help prevent the development of obesity and related metabolic and immunological disturbances (Fig. 4). To assess this question, HFD-fed mice were used 4,33-36 to measure several histological, immunological and metabolic biomarkers known to be associated with obesity and its adverse health effects. We show that RCI-1502 effectively regulates inflammation and reduces lipid accumulation caused by a sustained HFD regimen.

### The effects of RCI-1502 on HFD-associated weight gain in mice

The HFD obesity mouse model used in this study caused an increase in weight, development of hyperlipidemia, and alteration of pro-inflammatory markers 4,6,35,37-39. Our data in mice show that the weight gain induced by a HFD was significantly reduced by supplementing their diet with RCI-1502; the HFD also induced the regulation of serum biomarkers associated with obesity and CVD. These results agree with previous reports on liver 40 and cerebrovascular dysfunction 41-43 showing that high intake of omega-3 fatty acids found in fish oil 44 reduce HFD-induced weight gain in mouse models of obesity. Fish-oil extracts improve adipose tissue storage (dyslipidemia) and secretory functions of adipose tissue, and reduce insulin resistance, hepatic steatosis and inflammation 45. Furthermore, fat mass rates are decreased in rodents 46,47 and humans 48,49 consuming diets with fish oil (ω-3 FAEE) supplementation, probably due to mitochondrial-induced changes by these specific fatty acids 50. Adipose tissue, generated by a HFD, may modulate its effect in serum by providing intrinsic adiponectins, leptins and n-3 fatty acids. These findings are in line with animal model experiments conducted by our group 25-31 and others. RCI-1502 potentially blocks the excessive release of free fatty acids into the circulating serum, avoiding ectopic lipid deposition, thereby preventing obesity in mice.

The beneficial effects of RCI-1502 in the regulation of lipid homeostasis and obesity prevention were also analyzed with tests of motor behavior in all groups of mice. Our results align with previously recorded weights, where the pathophysiological progression of lipid accumulation due to HFD was efficiently prevented by RCI-1502 supplementation; this contributed to normal motor behavioral scores in RCI-1502-treated groups. The improved results in motor performance, observed in mice treated with RCI-1502 supplementation before HFD (Gr B), contrasted slightly with two other RCI-1502-treated groups (Gr A and C), and contrast markedly with HFD-fed mice (Gr E). All motor behavior and obesity measured parameters were strongly correlated, demonstrating a significant link between body weight gain and motor performance. These findings, similar to those described elsewhere 54,55, confirmed that overweight (obesity) differentially affects the risk for functional limitations and disabilities in motor performance and behavior 56.

### Effects of RCI-1502 on HFD-associated cardiovascular pathology in mice

The present study showed that RCI-1502 prevented the development of cardiovascular pathologies. Cardiovascular pathophysiological hallmarks are among the diagnostic criteria for metabolic syndrome, commonly associated with obesity, diabetes mellitus 57, chronic kidney disease and hypercholesterolaemia 58. Our data demonstrate that supplementing a HFD diet with RCI-1502 minimizes the pathological damage associated with the cardiovascular system, and prevents the development of cardiovascular lipid accumulation by acting as a lipoprotection agent. These findings agree with other reports that show that ω-3 fatty acid (fish oil) supplementation decreases lipoprotein triacylglycerol secretion 59, modulates serum levels of adiponectin and leptin 45 and improves several features of metabolic syndrome associated with CVD 44,60-62. Fish oil significantly reduces platelet aggregation, high-fat diet-induced steatosis, and serum lipid levels caused by eating a diet high in animal models. This reduction is due to the bioactivity of ω-3 fatty acids, specifically eicosapentaenoic acid (EPA) and docosahexahexaenoic Acid (DHA) that confer cardiovascular protection by blocking multiple atherogenic processes 62-66. Furthermore, dietary supplement of pure ethyl EPA and fish oil extract are effective antithrombotic agents by limiting platelet reactivity 67. Nevertheless, our study reveals for the first time that RCI-1502 clearly prevents the progression of atherosclerosis plaques as indicated by the specific biomarkers analyzed. In particular, VCAM-1 immunoreactivity was nearly tripled by HFD in the present study, and slightly detected in mice receiving a HFD supplemented with RCI-1502. The reduction of immunoreactivity to VCAM-1 in cardiovascular tissues from RCI-1502 fed mice, agrees with our previous reports 25, 29, since VCAM-1 is an early biomarker of vascular inflammation induced by atherosclerotic plaque accumulation 39. Progressive atheroma plaque accumulation may play a critical role as an initiator of obesity-associated inflammation, blood flow obstruction, peripheral thromboses, and myocardial infarction 68. Understanding what triggers the development of atheromas involves multiple factors such as lipoprotein content, blood sugar levels and hypertension 69. Our present data highlight the effect of fish-derived lipoproteins, such as leptin, as active agents against atheroma progression in animal models of obesity, as also reported elsewhere 70. Mice treated with RCI-1502 showed significant improvements in cardiovascular performance. This lipoprotein marine extract shows beneficial hypolipemic effects against the accumulation of lipid bodies in the cardiovascular system. Supplementation prior to HFD consumption provides lipo-cardioprotection to cardiovascular tissues via leptin bioactivity.

### Effects of RCI-1502 on HFD-associated liver pathology in mice

A high cholesterol-diet causes hypercholesterolemia. This lipid imbalance induces lipolysis and releases free fatty acids into the serum, where it is converted into triglycerides in the liver. If left untreated, this can lead to liver necrosis and cirrhosis. In this study, the liver was also analyzed since it is the first organ to metabolize ingested cholesterol and is therefore the main target of the lipoxidative damage caused by an imbalance between generated free radicals and an ineffective antioxidant defense mechanism 71.

Our findings are consistent with other studies that found lipid hepatotoxicity in HFD mice, resulting in severe steatosis and concomitant hepatocyte necrosis (data not shown) 72,73. The lipoprotective effect of RCI-1502 in the liver may be directly related to its bioactive properties such as regulation of fat accumulation, antiplatelet, absence of inflammatory and oxidative effects 44,66,72,74-76. Among the RCI-1502-treated groups, previous RCI-1502 administration induce a notable improvement in terms of hepato-lipidic degeneration, which is probably associated with the protective effects of intrinsic RCI-1502 fish-derived extract components. In particular, some of these components in the bioactive extract, such as fish-derived lipoproteins, have a crucial effect on fatty acids metabolism 77, 78, lipid homeostasis 79 and hepatic lipid accumulation 80,81, as well as an acute immunoregulatory role 29. Adiponectin is the most abundant lipoprotein secreted by adipocytes, and is a key component in the interrelationship between adiposity, insulin resistance and inflammation in the liver 82. RCI-1502 may alleviate dyslipidemia by stimulating phosphorylation and adenosin monophosphate kinase activation, exerting direct effects on vascular endothelium, decreasing the inflammatory response, and improving insulin sensitivity and glucose tolerance 83. Together, our findings confirm that adiponectin fish-derived lipoproteins prevent hepatosteatosis by reducing hepatic lipid accumulation through the regulation of lipogenesis and oxidative stress in obesity animal models 84.

### Effects of RCI-1502 on HFD metabolic syndrome in mice

Metabolic syndrome could constitute the previous stage to the development of CVD and diabetes mellitus. This study addresses how cardio-inflammatory processes, triggered by a high-fat diet, can be evaluated by analyzing blood biomarkers from a cardiovascular-related pre-clinical prediction panel. Specifically, 11 different groups of serum biomarkers were analyzed to evaluate their association with metabolic syndrome [urea, hs-RCP, cholesterol (COL, HDL, LDL), TG, HCY, Lpa, GOT, GPT and albumin] as these are the most analyzed inflammatory biomarkers in the CVD field. Our results suggest that the prevention of chronic metabolic disease by RCI-1502 may be mediated by an active anti-inflammatory action.

The increase in urea levels in serum, a catabolite of endogenous protein biomarker 85, correlates directly with high levels of toxic nitrogen metabolites, produced during the catabolism of proteins and amino acids, indicating a metabolic disorder 86. Mice in HFD/CS groups A and B showed similar levels of urea in serum as controls (Diet) and lower levels than group C and HFD-fed mice. The high levels of urea in HFDb/CS group A, compared to controls, may be due to the metabolic disorder effect of the HFD period prior to RCI-1502 supplementation, triggering the accumulation of this endogenous catabolite. Serum creatinine is a critical indicator of renal health 87; creatinine blood levels rise when filtration in the kidney is impaired. All HFD/CS-treated mice showed similar levels of serum creatinine to control groups (Diet), and lower levels than did HFD-fed mice. The high serum levels of creatinine in the HFD-fed mice, compared with controls and HFD/CS, suggest an increased likelihood of developing renal disorder or systemic readjustment due to HFD-induced metabolic effects, reinforcing the modulating effect of RCI-1502 against these adverse events. In the past decades, compelling evidence support the reduction of total cholesterol (TC) and LDL as effective in preventing CVD events 88. Moreover, cholesterol meta-analysis reports confirmed the dose-dependent reduction in CVD morbidity and mortality achieved by lowering LDL levels in blood serum 89. All HFD/CS-treated mice showed similar pattern levels of cholesterol biomarkers in serum to control groups (Diet), while HFD mice showed the opposite pattern, where TC, LDL and TC/HDL display high concentration levels. The absence of hypercholesterolemia in mice from groups A-C, compared to HFD-fed mice group, may be due to the modulating effect of RCI-1502 supplementation; RCI-1502 may prevent the accumulation of cholesterol in the vascular system, suggesting that it may act as a lipoprotective agent against atherosclerosis risk and CVD. Triglycerides, are significant and independent risk factors for CVD 90. A typical CVD pattern in patients are elevated triglyceride levels accompanied by low HDL cholesterol 91. All HFD/CS-treated mice showed lower levels of triglycerides in serum than control groups (Diet), whereas HFD mice had high levels of triglycerides. The most significant difference was observed in groups A and C, probably due to the modulating effect of RCI-1502 supplementation against the two main sources of plasma triglycerides: exogenous (i.e., from dietary fat) and endogenous (from the liver), carried in very-low-density lipoprotein (VLDL) particles. RCI-1502 supplementation in a HFD diet prevents increases in serum triglyceride concentrations that contribute to an increased risk of CVD. Such increases are linked to obesity, metabolic syndrome, proinflammatory, pancreatitis, and prothrombotic biomarkers. High levels of hyper-homocysteine (HCY) in serum induce the expression in vascular cells of numerous proinflammatory agents such as MCP-1, IL-8, VCAM-1, E-selectin, and CXCL16 and its receptor CXCR6155 94,95. HCY levels were lower in all HFD/CS-treated animals than in controls (Diet), with HFD mice exhibiting high HCY concentrations. Among HFD/CS-treated mice, the most significant difference was observed in mice from group A. This reduction may probably be due to the regulating effect of RCI-1502 in response to vascular inflammation and endothelial dysfunction. Meta-analysis studies showed that lipoprotein (a), an independent proatherogenic and prothrombotic risk factor, was directly associated with incident coronary heart disease and ischemic stroke 96. Mice from HFD/CS groups A and B showed similar levels of lipoprotein (a) in serum to control group (Diet) and significantly lower levels than group C and HFD-fed mice. When compared to controls, HFDba/CS mice (group A) had the lowest levels of the lipoprotein biomarker; this may be attributed to the extensive therapy period with RCI-1502, which prevented endogenous vascular buildup of LDL-cholesterol and platelet accumulation. Concentrations of hypersensitive C-reactive protein (hs-CRP), a serum biomarker of inflammation, are elevated in response to tissue injury and atherosclerotic CVD risk 97,98. In patients, HS-CRP levels rapidly increase as a result of stroke 99, although, a wide range of inflammatory conditions may also trigger this response. All HFD/CS-treated animals had CRP levels in their serum that were comparable to control groups (Diet) but lower than HFD-fed mice. High hs-CRP serum levels in HFD-fed animals, compared to controls and HFD/CS-treated mice, suggest a greater risk of developing CVD or ischemic events, which support the modulating effect of RCI-1502 against these metabolic events. HS-CRP levels in HFDba/CS-treated mice were similar to control levels, while mice from the other HFD/CS-treated groups showed slightly higher levels, but not as high as in HFD-fed mice. Therefore, the modulating effect of CS may be due to its extensive period of supplementation, regulating vascular and hepatic lipid metabolism. Blood GOT activity represents the capacity of brain glutamate degradation, and is an efficient and novel neuroprotective tool against ischemic stroke 100. All HFD/CS-treated mice had lower levels of GOT in serum than control groups (Diet), while HFD mice had very high GOT concentrations. Among the HFD/CS-treated mice, the most significant difference was observed in groups A and B, probably due to the modulating effect of RCI-1502 in glutamate metabolism. The GPT is a valuable screening biomarker to detect hepatic necrotic processes, such as asymptomatic viral hepatitis and non-alcoholic fatty liver disease. Additional factors that affect serum GPT levels include body mass index, total cholesterol and triglyceride levels 101. HFD/CS-treated mice showed similar levels of serum GPT as controls (Diet), while HFD mice had very high concentrations of GPT. These data may indicate that RCI-1502 supplementation regimen positively affects hepatic metabolism, even when altered by a HFD, suggesting a preventive effect by RCI-1502 on hepatic injury. Ischemia-modified albumin is a biomarker for CVD; high levels of albumin is associated with cardio-metabolic risks and may be a sign of microvascular dysfunction in individuals with metabolic syndrome 102. HFD/CS-treated mice had slightly higher levels of serum albumin than controls (Diet), while HFD mice had high concentrations of serum albumin. This pattern was expected since this biomarker also assesses nutritional status, and is affected by the high-fat composition of the supplementation. However, HFD/CS mice showed similar levels of serum albumin compared with control mice, indicating a metabolic regulatory function of RCI-1502. The total antioxidant capacity was measured by analyzing small antioxidant molecules in serum 103. Oxidative stress, in the development of visceral adipose tissue, subclinical atherosclerosis and metabolic syndrome are well-known 104. All HFD/CS-treated mice showed significantly high serum levels of TAS versus control groups (Diet), while HFD-fed mice had very low levels of TAS. Among the HFD/CS-treated groups, the most significant difference was detected in group A; these mice had strongly elevated TAS levels, probably due to the antioxidant induction effect of RCI-1502 through its capability to counteract reactive oxygen species (ROS).

Taken together, the present study found that RCI-1502 drastically reduced the adverse effects of a HFD by significantly modulating the main cardiovascular biomarkers in serum such as LDL, HDL, CT, TG or hs-RCP, among others (see Table 4). Fat-modified diets reduced total cholesterol and triglyceride levels, but there was no evidence of effects on weight, body mass index, LDL or HDL cholesterol that contributes to the development of metabolic syndrome 105. However, by adding RCI-1502 to a HFD, classical CVD risk factors were regulated within normal control levels, which improved the body mass index, renal function, hepatic metabolism and antioxidant status.

## Conclusions

Dietary high-fat content can cause obesity by activating various metabolic processes, which can alter fat oxidation and deposition rates, resulting in metabolic syndrome and macrovascular complications. Correction of saturated fat consumption (hypertriacylglycerolemia) in combination with hypolipemic lipoproteins (leptin, adiponectin) may lower the risk of CVD in obese individuals. A sardine-derived bioproduct (RCI-1502) reduces or prevents metabolic disturbances associated with the development of obesity in HFD mice models by regulating lipid homeostasis, particularly by acting against systemic inflammation, hepatic steatosis, and cardiovascular lipid accumulation. More research is required to better understand the molecular metabolic mechanisms involved, particular the role of lipoproteins in altering metabolic signaling after food supplementation with fish extracts.

## Supplementary Material

Supplementary graphs.

## Institutional Review Board Statement

All experimental procedures were performed in accordance with the European Community Law (86/609/EEC), European Union Directive 2016/63/EU and the Spanish Royal Decree (R.D. 1201/2005), and following the approval of the Ethics Committee of the EuroEspes Research Center.

## Author Contributions

Conceptualization, methodology and validation, R.C. and I.C.; Investigation, resources, data curation and writing—original draft preparation I.C., L.C., O.M.I. and V.N; Software and writing—review and editing I.C., V.N. and L.C. All authors have reviewed the manuscript and have agreed with all of the contents.

## Figures and Tables

**Table 1:**
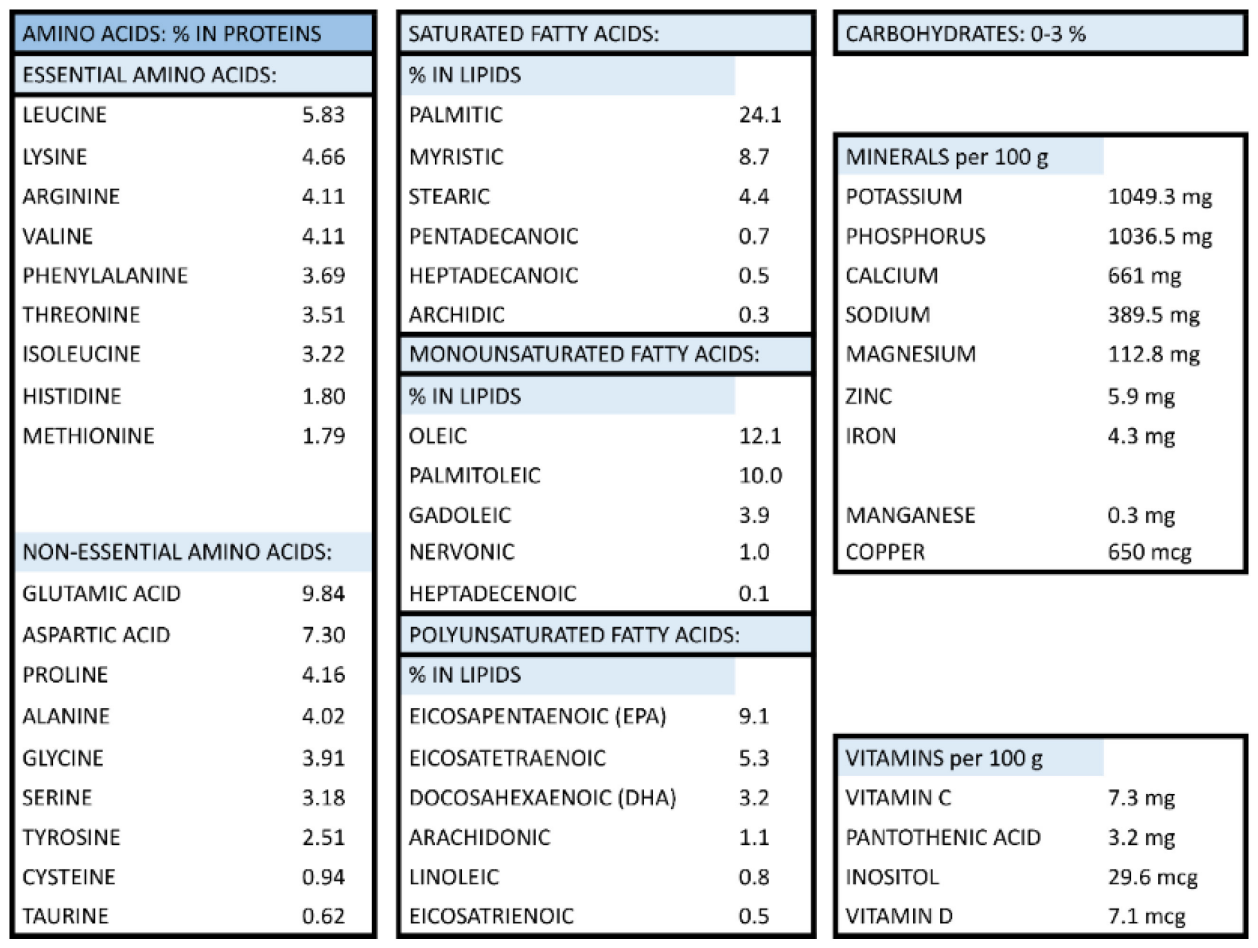
Composition of RCI-1502 (sardine derived extract)

**Table 2:** Mouse diet/supplementation regimen

	Weeks of supplementation							
	1	2	3	4	5	6	7	8
Gr A (HFDba/CS)	HFD	HFD	CS	CS	CS	CS	HFD	HFD
Gr B (HFDa/CS)	D	D	CS	CS	CS	CS	HFD	HFD
Gr C (HFDb/CS)	HFD	HFD	CS	CS	CS	CS	D	D
Gr D (D/CS)	D	D	CS	CS	CS	CS	D	D
Gr E (HFD)	HFD	HFD	HFD	HFD	HFD	HFD	HFD	HFD
Gr F (Diet)	D	D	D	D	D	D	D	D

(HFD, High Fat Diet; D, regular Diet; CS, RCI-1502 supplemented diet).

**Table 3:** The nutritional composition of the diets per 100 gr provided to mice

	Normal (control) diet		High-fat diet		RCI-1502	
	(g)	(% kcal)	(g)	(% kcal)	(g)	(% kcal)
Protein	18.6	20	10.2	11	67.4	72
Carbohydrates	68.2	75	28.3	31	2.6	3
Fats	4.4	5	56.7	58	23.4	25
*Saturated*	(2)	-	(18.6)	-	(7.9)	-
*Monosaturated*	(1.3)	-	(4.1)	-	(4.5)	-
*Polyunsaturated*	(1.1)	-	(4)	-	(11)	-
Total	91.2	100	95.2	100	93.4	100
Energy (kcal/g)	3.89		5.14		4.9	

**Table 4:**
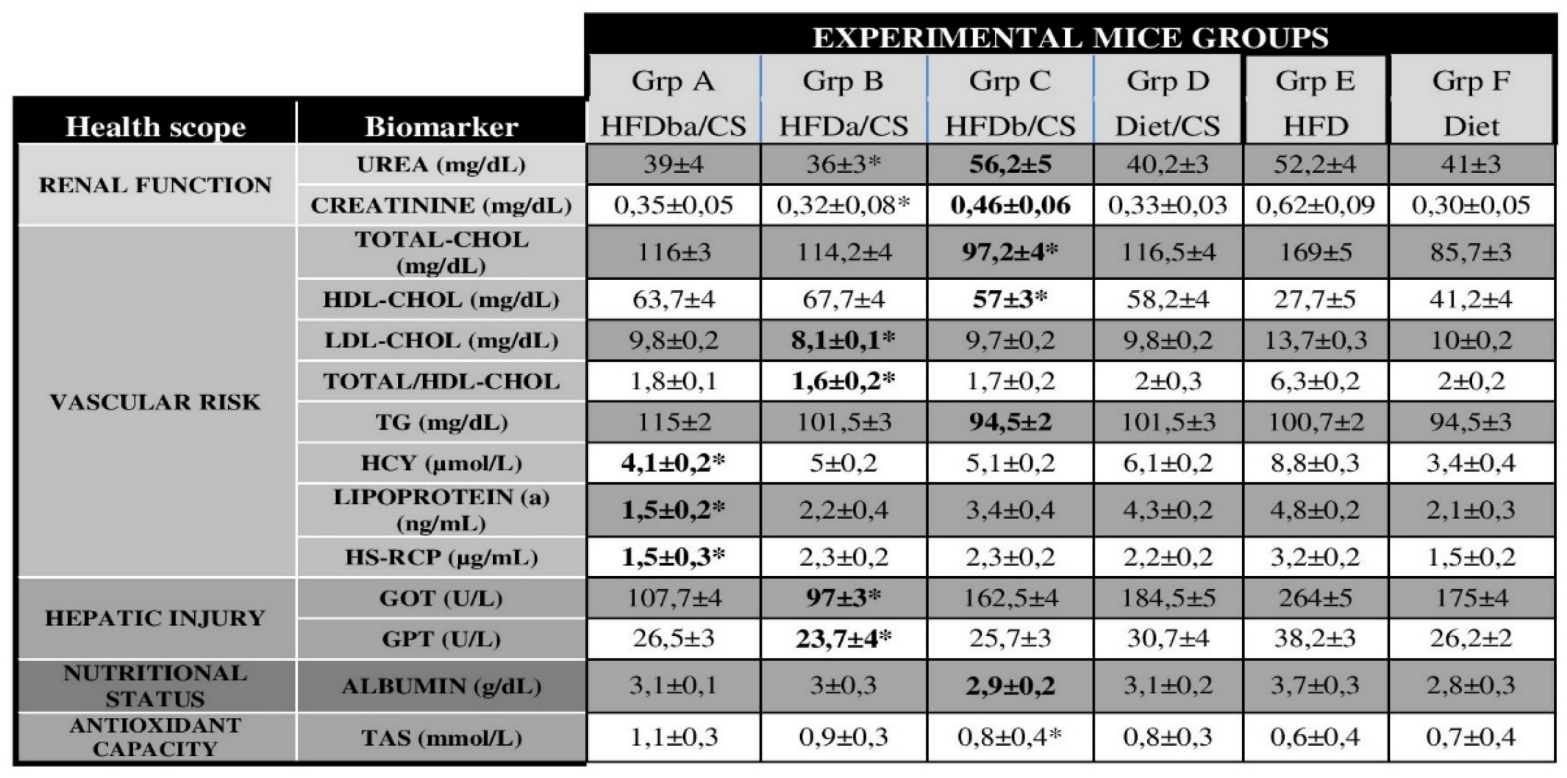
Serum levels of cardiovascular biomarkers in experimental treated mice. In bold, the biomarker values with notable differences between RCI-1502 treated mice and HFD-treated mice. Asterisks (*), represent significant differences between the experimental groups, (p < 0.05). Data are means ± SEM.

## Abbreviations

ALB: Albumin
CHD: coronary heart disease
COL: cholesterol
hs-CRP: hypersenstive reactive C protein
CS: RCI-1502
CVD: cardiovascular disease
CXCL16: inflammatory chemokine
DHA: docosahexaenoic acid
DHA: docosahexaenoic acid
EPA: eicosapentaenoic acid
EPA: eicosapentaenoic acid
FLD: fatty liver disease
GOT: Glutamate oxaloacetate transaminase
GPT: Glutamine-pyruvate transaminase
HCY: homocysteine
HDL: high-density lipoprotein
HE: hematoxylin and eosin
HFD: high-fat diet
LDL: low-density lipoprotein
LE: LipoEsar
MetS: metabolic syndrome
OM3FAs: polyunsaturated omega-3 fatty acids
TAS: Total antioxidant capacity
TBS: Trizma buffered saline
TC: total cholesterol
TG: Triglycerides
VCAM-1: vascular inflammation biomarker
VLDL-TG: very low density lipoprotein triacylglycerols
ω-3 FAEE: omega-3 fatty acid ethyl ester
