# Supplementary material for: Cardiovascular and lipid-lowering effects of a marine lipoprotein extract in a high-fat diet-induced obesity mouse model
Source: Int J Med Sci. 2023 Jan 22;20(3):292–306. doi: 10.7150/ijms.80727 (PMC9969509; doi:10.7150/ijms.80727)
Supplement: Supplementary file 1 — Supplementary graphs. [file ijmsv20p0292s1.pdf]

Manuscript: *IJMS*

Supplementary data

Graphs showing that HFD and control groups reach similar obesity biochemical parameters at early weeks of treatment with RCI-1502 (CS):

Graph 1. Baseline-obesity timeline in HFD mice: Body weight

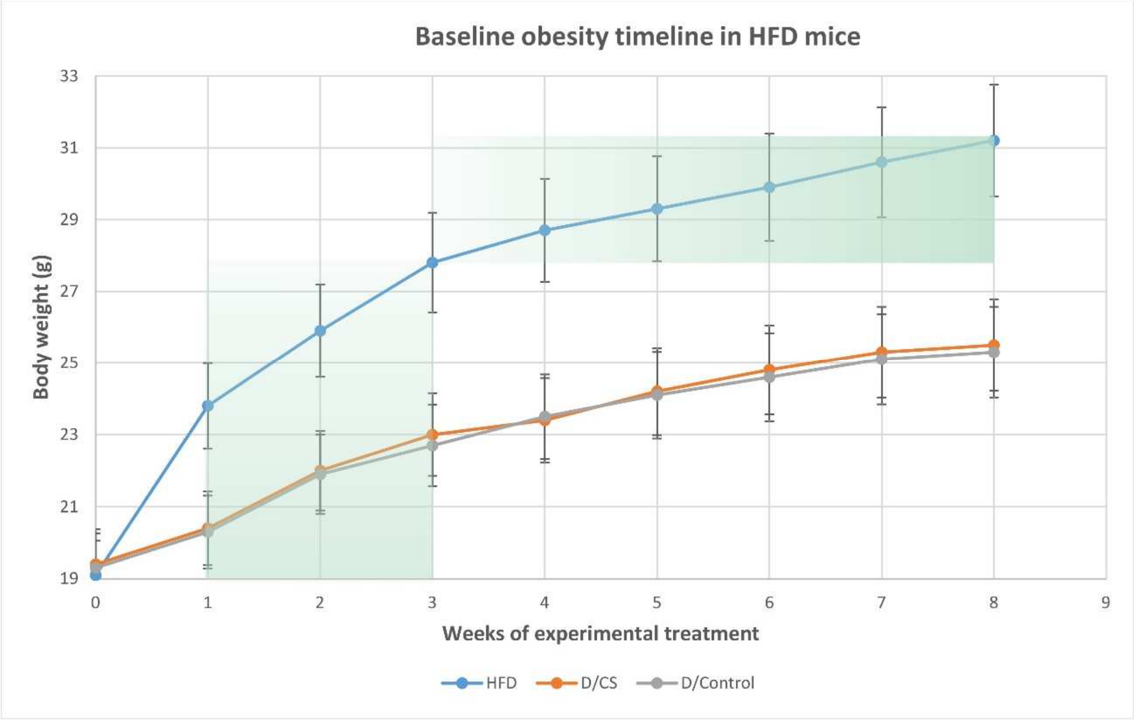

Graph 2. Baseline-obesity timeline in HFD mice: Abdominal fat

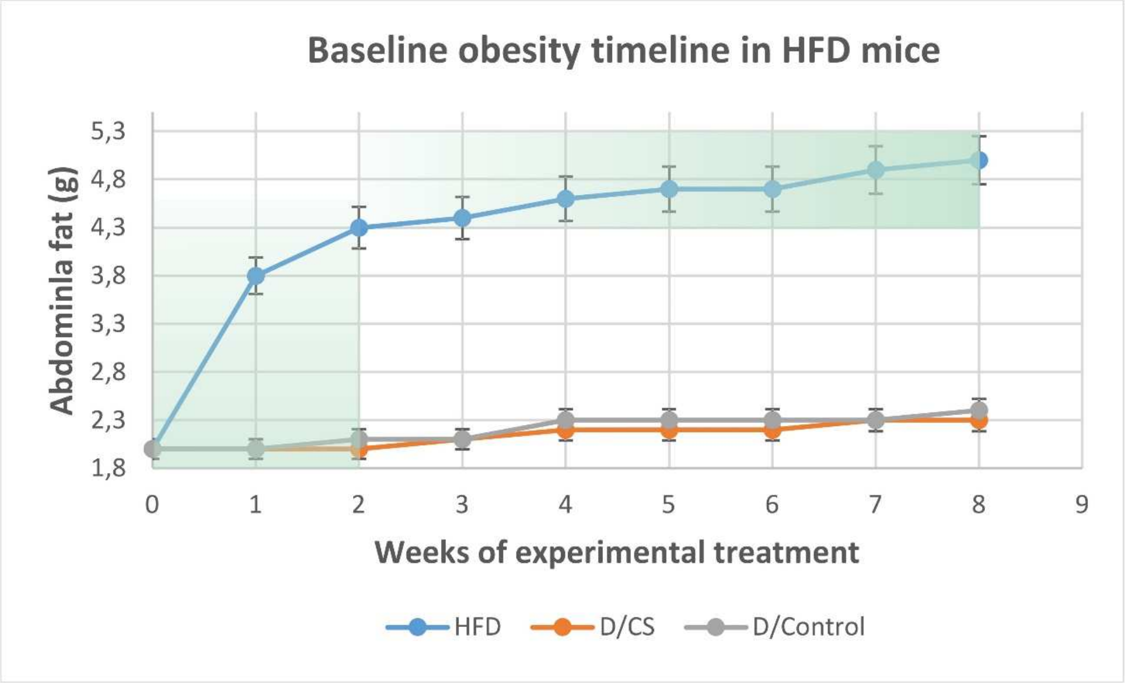

Graph 3. Baseline-obesity timeline in HFD mice: Triglycerides

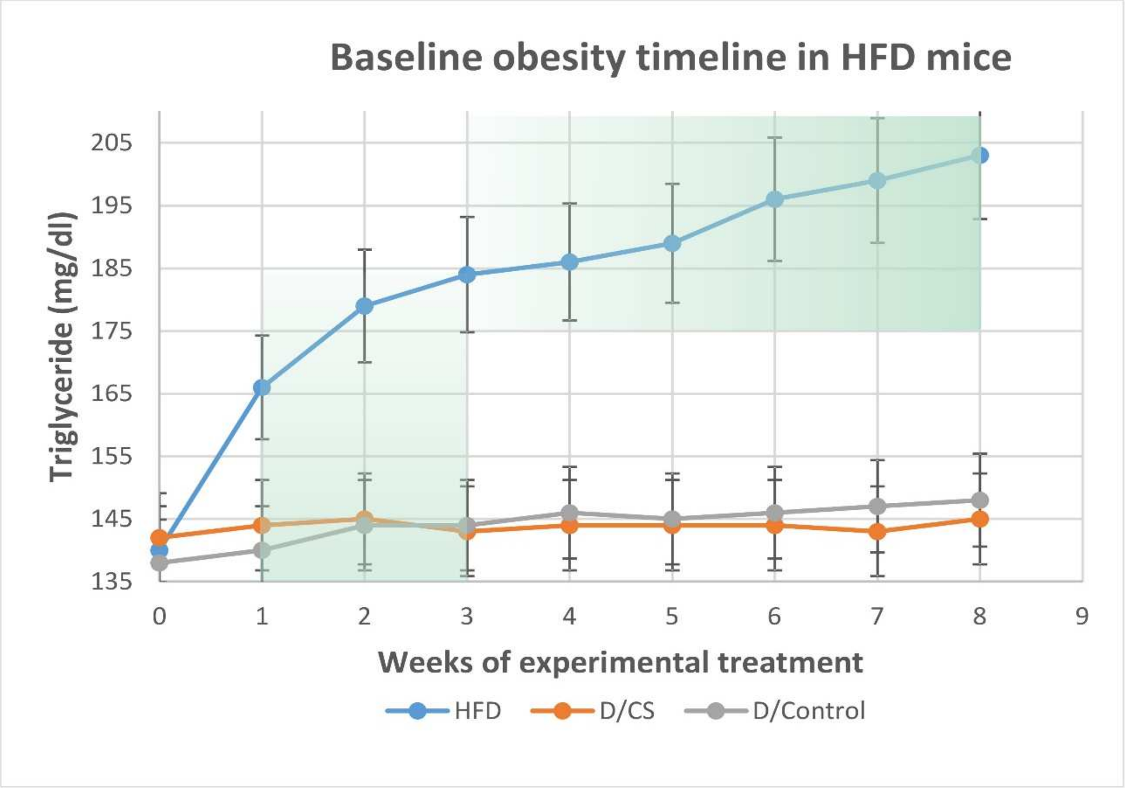

Graph 4. Baseline-obesity timeline in HFD mice: Total Cholesterol

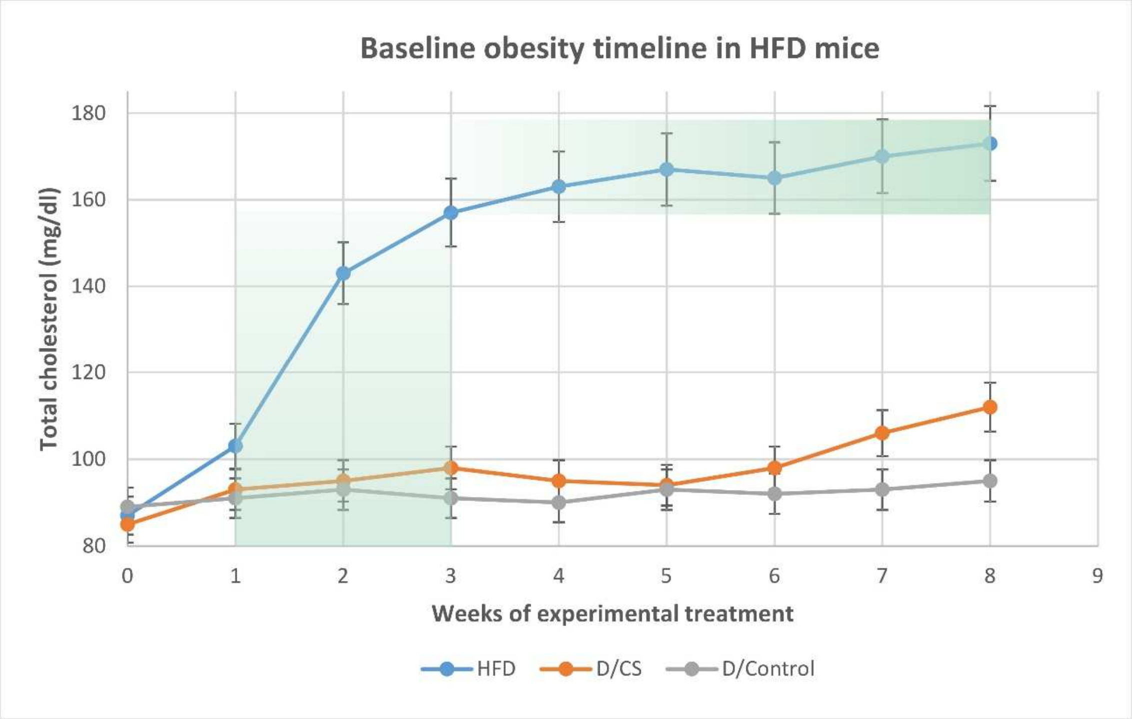

Graphs 1-4: Physiological and Biochemical parameters in control and HFD-induced mice showing that they reach similar obesity levels at early weeks of treatment. (1) Direct correlation of HFD with increasing body weight compared to controls, as early as 3 weeks of treatment. (2) Progression of abdominal fat weight of HFD and control groups. HFD group reaches similar obesity levels after 2 weeks of treatment. (3) Serum triglyceride parameters during the 8 weeks of treatment, showing that HFD group reaches similar obesity levels at 2 weeks of treatment. (4) Serum total cholesterol parameters (HDL/VLDL) in all groups, where HFD reaches similar obesity levels at 3 weeks of treatment. Data represent the means ± SEM of physiological and biochemical parameters per treatment group. Note that green shadow zones indicate increased cholesterol levels (vertical green zones) to reach the obesity parameters (horizontal green zones).
